# Prevalence of depression in junior and senior adolescents

**DOI:** 10.3389/fpsyt.2023.1182024

**Published:** 2023-12-11

**Authors:** Jing Zhang, Dehuan Liu, Linwei Ding, Guankui Du

**Affiliations:** ^1^Hainan Provincial Anning Hospital, Haikou, China; ^2^Hainan Provincial Bureau of Human Resources Development, Haikou, China; ^3^Institute of Gut Microecology and Health, Hainan Medical University, Haikou, China; ^4^Department of Biochemistry and Molecular Biology, Hainan Medical University, Haikou, China; ^5^Biotechnology Laboratory, Hainan Medical University, Haikou, China; ^6^Department of Breast Surgery, The First Affiliated Hospital of Hainan Medical University, Haikou, China

**Keywords:** latent class analysis, PHQ-9, depression, adolescent, high school, junior middle school, vocational school

## Abstract

**Background:**

Depression affects the development of adolescents and makes it difficult for them to adapt to future life. The purpose of this study was to elucidate the population characteristics of adolescent depression.

**Methods:**

This study measured depression based on the Patient Health Questionnaire-9 items and sociodemographic questionnaire. A total of 8,235 valid questionnaires were collected from six schools in Haikou and Qionghai, Hainan Province, covering the ages of 13 to 18. The questionnaires included high schools with multiple levels, including general high schools, key high schools, and vocational high schools. Latent category analysis (LCA) was used to identify potential categories of depressive symptoms among adolescents. Latent Class Analysis (LCA) was used for determining depressive symptom latent categories and their proportional distribution among adolescents.

**Results:**

LCA analysis divided the data into 3 categories, namely no depression, low depression, and high depression groups. The percentage of the high depression group was 10.1%, and that of the low depression group was 48.4%. The Jorden index was greatest for a PHQ-9 score of 14.5. The 1^st^ grade of junior middle school students entered the high and low depression groups 1.72 and 1.33 times more often than seniors. The number of the 1^st^ grade of high school students included in the high and low depression groups was 1.55 and 1.42 times of the 3^rd^ grade of high school students group. The detection rate of the high depression group of vocational school adolescents was 13.5%, which was significantly higher than that of key high schools (9.6%) and general high schools (9.0%).

**Conclusion:**

This study found that 1^st^ grade of junior middle school students and the 1^st^ grade of high school students were more likely to fall into depressive conditions. Moreover, Adolescent girls require more attention than boys. Vocational school students need more psychological guidance.

## 1 Introduction

Adolescents are a high-risk group for depression, with approximately 20% of 10-to 20-year-olds experiencing it. Depression can negatively affect the adolescent’s development and lead to challenges adapting to their future life (1). Adolescent depression is manifested in problem behaviors such as risky sexual behavior, substance abuse, delinquency, self-harm, suicide, low attendance, poor academic performance, and dropping out of school (2, 3). Between 2009 and 2019, the prevalence of major depressive episodes among adolescents aged 12–17 years in the United States increased from 8.1 to 15.8% (4). One study investigated 4,100 Chinese students aged 11–16 years and found a high prevalence of depression of 34.0% (5). A meta-analysis involving 144,000 Chinese adolescents detected a prevalence of depression of 24.3% (6). Accurate access to trend data on the prevalence of depression in adolescents can provide data to support depression interventions.

Sex differences emerge at puberty in response to increased gonadotropin pulses. During prepuberty, gender differences in the rates of detection of internalizing problems such as depression, anxiety, and eating disorders are not significant, with slightly higher rates for boys and nearly equal rates for boys and girls. Upon entering early adolescence, the rate of detection of internalizing problems increases in girls and is approximately double that of boys (1, 7, 8). However, there are also studies that did not find gender differences in the prevalence of depression in adolescence (6). In addition, a recent study had shown a gradual increase in the prevalence of depression from 1^st^ grade of junior middle school students to the 3^rd^ grade of high school students (6). Another study indicated that approximately 33.4 and 28.8% of adolescents exhibited depressive symptoms in Grades 7 and 9, respectively (9). Opinions differ on which grade level has a higher prevalence of depression. Thus, the characterization of depressive symptoms in adolescents across grade levels and genders requires the acquisition of accurate data for analysis and is expected to provide a framework for psychological intervention strategies for adolescent children.

Adolescents in different environments encounter different types of pressures. Structural factors such as lack of opportunities and other resources are considered to be risk factors for adolescent depression (10). Middle school students in China often face exams to advance from middle school to high school, while high school students face exams to advance from high school to university. In the middle school to high school entrance examination, students with higher grades have more opportunities to enter key high schools, which means they have a greater chance of entering good universities. Students with average grades can attend regular high schools and have a greater chance of entering regular universities and vocational colleges. Students with poor grades can only attend secondary vocational schools, which may result in the need to enter the job market after three years. Studies have shown that academic stress is an important factor leading to depression in adolescents (11). It can be inferred that academic pressure is one of the main pressures faced by students in key high schools and ordinary high schools, while employment pressure and poor learning environment may be the main pressures faced by students in secondary vocational schools. Therefore, the level of depression occurrence and influencing factors among students in different environments are worth in depth analysis.

The detection rate of adolescent depression varied widely among studies, and the instruments used and the detection criteria influenced the interpretation of the results. Tang et al. [6]used the depression factor score in the SCL-90 as a defining criterion and found a highly variable detection rate of 5.1%, with a cut-off value of 3. The detection rate was 24.9% with a cut-off value of 2. Latent Profile Analysis has been used in many studies to explore unobserved subgroups (12, 13). Chen, Huang, Yu, and Review (14) divided adolescent depressive symptoms into 3 subgroups using latent profile analysis. Ling et al. (15) used Latent Class Analysis (LCA) to classify adolescent depressive disorder into 5 subgroups, with 8.2% in the “probable clinical diagnosis of depression group.” The method of potential profile analysis can circumvent the problem of inconsistent criteria for determining the critical score of the scale. Therefore, this study explored the use of latent profile analysis for subgroup analysis of depressive symptoms in adolescents aged 13–18 years.

In this study, six schools covering six levels of middle and high school were selected for the analysis of adolescent depression, and stratified analysis was conducted based on age, gender, and environmental characteristics.

## 2 Methods

### 2.1 Participants

The cities of Haikou and Qionghai in Hainan Province were in the process of improving the construction of a psychosocial service system, and a great deal of work had been carried out around the mental health of adolescents. Based on the principle of random sampling, six schools were selected in Haikou and Qionghai. A total of 8,235 valid questionnaires were collected, covering adolescents aged 13 to 18. The questionnaire also included multiple levels of high schools, including general high schools, key high schools, and vocational high schools. To examine the differences between gender and grade level in depressed mood, all data were included in the analysis to form the first cohort (5,382 validated questionnaires); to examine the effect of school category on depressed mood, the second queue (2,853 validated questionnaires) data was merged into the first queue (Table 1).

**Table 1:** Description of psychosocial variables related to participants.

	First data queue (*N* = 5,382) *N*%		Second data queue (*N* = 2,853) *N*%	
Variables				
Gender				
Male	2,442	45.4	1,149	40.3
Female	2,940	54.6	1704	59.7
Grade				
1^st^ grade of junior middle school students	634	11.8%	-	-
2^nd^ grade of junior middle school students	616	11.4%	-	-
3^rd^ grade of junior middle school students	561	10.4%	-	-
the 1^st^ grade of high school students	1,131	21.0%	1,131	39.6%
2^nd^ grade of high school students	1722	32.0%	1722	60.4%
3^rd^ grade of high school students	718	13.3	-	-
School type				
Vocational high school	-	-	1,023	35.9%
Key high school	-	-	1,109	38.9%
Ordinary high school	-	-	721	25.3%

### 2.2 Measures and procedure

The Patient Health Questionnaire-9 Item (Chinese version; PHQ-9) is a 9-item self-report measure of depression (16). Responses are captured over the past two weeks and rated on a four-point Likert scale ranging from 0 (not at all) to 3 (almost every day). The total score ranges from 0 to 27, with a minimum score of 0 and a maximum score of 27. The Cronbach’s alpha coefficient was 0.835 in the present study.

This study used a psychometric software system to collect data, and data with a response time of fewer than 14 s were excluded. The sociodemographic questionnaire consisted of questions regarding students’ age, gender, name of school, and grade.

### 2.3 Data analysis

The latent category analysis was conducted using Mplus 8.3 software, which was used to determine the latent depression symptom categories and their proportional distribution among adolescents. The unordered multifactorial logistic regression analysis was conducted using SPSS 22.0, which examined the predictive effects of predictor variables on the potential depression categories of adolescents. Additionally, a stratified Chi-square test was used to analyze the interaction of grade and gender on depression detection rates.

The Akaike Information Criterion (AIC), Bayesian Information Criterion (BIC), and Sample Size-adjusted BIC (aBIC) are used to assess model fit by comparing the difference between the expected value and actual value. A smaller value represents a better fit. According to Lubke and Muthén (17), an entropy of less than 0.60 corresponds with more than 20% of individuals having classification errors, and an entropy of more than 0.80 indicates a higher classification reliability. Additionally, larger sample sizes and lower entropy values are associated with lower classification accuracy, regardless of the number of categories (12). The BLRT and LMR are used to compare the fit differences between the k-1 and k-categorized models. The significant BLRT *p*-values and LMR p-values indicate that the k-categorized model is better than the k-1 category (13). Sometimes, the BIC value decreases monotonically with the number of categories, but there is never a minimum value. The steep-slope method of factor number determination in EFA may be of interest. This is used to determine the appropriate model (18).

In the unordered logistic regression analysis, data from the first cohort were analyzed to examine the predictive effects of grade and gender on depression. The latent category was grouped using the Likert scale as a dependent variable. Grade and gender were used as variables in the univariate regression analysis. The pre-and postadjustment parameters were used to determine the inclusion of variables. The variables with a relative change in the odds ratio of more than 10% were ultimately selected for inclusion in the equation (19).

The stratified Chi-square test was used to compare the detection rates in high and low depression groups, with a grade as a between-group variable and sex as a within-group variable. The Chi-square test was performed as a *post hoc* test using the Bonferroni method.

Jorden index calculation method. According to the formula “Jorden index = Se + Sp-1,” the point with the largest index for all possible critical values was the best critical value for the PHQ-9 scale calculated using this procedure (20).

## 3 Results

### 3.1 Latent category analysis of depressive status in adolescents aged 13–18 years

As shown in Table 2, the single-categorization model was utilized as the baseline reference point. Each time, a single latent category was added to form a total of five latent categories. As the number of categories increased, AIC, BIC, and aBIC decreased sequentially. However, the decrease of AIC, BIC, and aBIC slowed down for the 4-category model and 5-category model. The LMR and BLRT tests showed that all models reached a significant level. All classification models had an entropy value above 0.82, with the 3 classification models having the highest entropy values. Therefore, the 3-category model was selected for the subsequent analysis.

**Table 2:** Potential class analysis model evaluation indicators.

Model	K	AIC	BIC	aBIC	LMR	BLRT	Entropy	Profile prevalence				
								1	2	3	4	5
1C	28	111220.12	111404.66	111315.69	-	-	1.000	100				
2C	57	96476.35	96852.03	96670.90	0.000	0.000	0.871	51.6	48.3			
3C	86	91080.44	91647.25	91373.97	0.000	0.000	0.903	10.1	48.4	41.5		
4C	115	89679.40	90437.34	90071.91	0.000	0.000	0.851	9.94	27.3	24.7	38.1	
5C	144	88387.62	89336.70	88879.11	0.000	0.000	0.825	14.4	9.92	21.6	29.7	24.4

Three potential categories were classified based on the different mean and total score means of each PHQ-9 item. Category C1 was labeled as the “high depression” group, category C2 was labeled as the “low depression” group, and category C3 was labeled as the “no depression” group (Table 3). The average attribution probability of each category to itself ranged from 0.948 to 0.995, and the attribution error rate of each category ranged from 0.000 to 0.052.

**Table 3:** Average attribution probability for each potential class.

Category lanten profile	Profile membership			M(SD) total score for the depression
	C1 (*n* = 544)	C2 (*n* = 2,605)	C3 (*n* = 2,233)	
C1	0.995	0.005	0.000	18.209(3.28)
C2	0.007	0.953	0.040	8.75(2.76)
C3	0.000	0.052	0.948	1.77(1.74)

The Se, Sp, and Yordon indices were calculated for the total score of the PHQ-9 (Table 4). The Jorden index was the highest for a score of 14.5, which can be considered a high depression threshold.

**Table 4:** Se, Sp, and Jorden index of PHQ-9 total score at each threshold value.

PHQ-9 score	Se	1-Sp	Yoden index
14.50	0.98	0.00	0.98
13.50	0.99	0.03	0.96
12.50	0.99	0.06	0.93
11.50	1.00	0.10	0.89
10.50	1.00	0.15	0.85

### 3.2 Characteristics of depressive symptoms in adolescents aged 13–18 years

**Figure 1:**
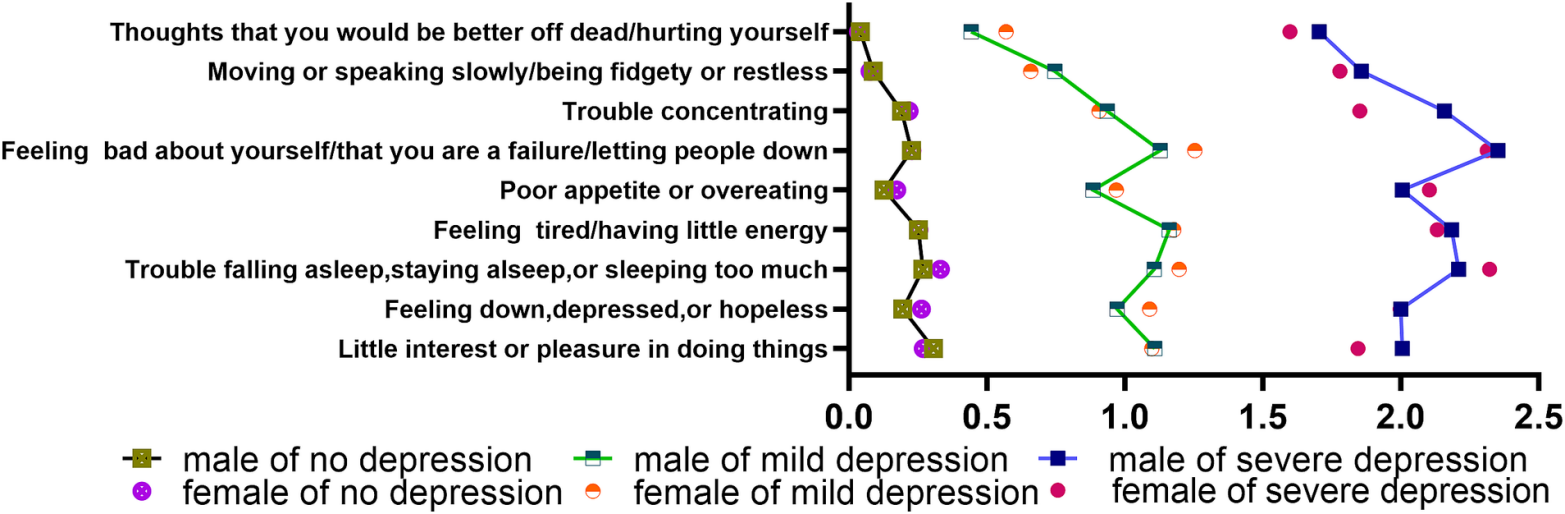
Characteristics of depressive symptoms in adolescents.

As shown in Figure 1, the high depression group had four entries with a mean score greater than 2, including “Feeling bad about yourself/that you are a failure/letting people down (male: M = 2.35, SD = 0.76; female: M = 2.31, SD = 0.80) “, “Trouble falling asleep, staying asleep, or sleeping too much (male: M = 2.21, SD = 0.84; female: M = 2.32, SD = 0.82) “, “Feeling tired/having little energy (male: M = 2.18, SD = 0.85; female: M = 2.13, SD = 0.77) “, and “Poor appetite or overeating (male: M = 2.01, SD = 1.00; female: M = 2.10, SD = 0.87) “. There were 2 entries in the high depression group with significant gender differences and higher scores for boys than girls, namely “Little interest or pleasure in doing things (male: M = 2.01, SD = 0.86; female: M = 1. 85, SD = 0.82) “and “Difficulty concentrating (male: M = 2.16, SD = 1.00; female: M = 1.85, SD = 0.98) “.

Both the low depression and high depression groups exhibited similar curve scores for nine items. Five items had a mean score above 1, including “Feeling bad about yourself/that you are a failure/letting people down,” “Feeling tired/having little energy,” “Having trouble falling asleep, staying asleep, or sleeping too much,” and “Feeling down, depressed, or hopeless.” Two of these items demonstrated significant differences between boys and girls, namely “Feeling bad about yourself/that you are a failure/letting people down (male: M = 1.13, SD = 0.82; female: M = 1.25, SD = 0.79) “and “Trouble falling asleep, staying asleep, or sleeping too much (male: M = 1.11, SD = 0.85; female: M = 1.20, SD = 0.85) “.

### 3.3 Analyzing differences in depressed mood according to grade and gender

An unordered multinomial logistic regression analysis was conducted using the potential category of depressive mood in adolescents as the dependent variable (Table 5). The detection rate of depression was significantly higher in the 1^st^ grade of junior middle school and the 1^st^ grade of high school students compared to the C3-no-depression group. The 1^st^ grade of junior middle school students entered the high and low depression groups 1.72 and 1.33 times more frequently than seniors, respectively. The number of the 1^st^ grade of junior middle school students included in the high and low depression groups was 1.72 and 1.33 times that of the 3^rd^ grade of high school group, respectively. The number of the 1st grade of high school students included in the high and low depression groups was 1.55 and 1.42 times that of the 3^rd^ grade of high school group, respectively. Meanwhile, the number of 3^rd^ grade of junior middle school students included in the high and low depression groups was 0.29 and 0.54 times that of the 3^rd^ grade of high school group, respectively. Therefore, the 1^st^ grade of junior middle school is the grade with the highest incidence of depression.

**Table 5:** Unordered multifactorial logistic regression of gender and grade on potential class.

Variables		Severe depression (*N* = 615)							Mild depression (*N* = 2,655)					
	B	SE	Wald	P	OR	CI (95%)		B	SE	Wald	P	OR	CI (95%)	
gender(female)male grade(3^rd^ grade of high school) 1^st^ grade of junior middle school 2^nd^ grade of middle school 3^rd^ grade of middle school 1^st^ grade of high school 2^nd^ grade of high school	−0.62	0.10	37.68	0.00	0.54	0.44–0.65		−0.50	0.06	69.26	0.00	0.61	0.54–0.68	
	0.54	0.21	6.83	0.01	1.72	1.15–2.59		0.28	0.13	5.17	0.02	1.33	1.04–1.70	
	−0.39	0.26	2.27	0.13	0.68	0.41–1.13		−0.45	0.16	7.95	0.00	0.64	0.46–0.87	
	−1.23	0.30	17.19	0.00	0.29	0.16–0.52		−0.62	0.16	14.57	0.00	0.54	0.39–0.74	
	0.44	0.19	5.35	0.02	1.55	1.07–2.25		0.35	0.11	10.22	0.00	1.42	1.15–1.77	
	0.04	0.18	0.04	0.85	1.04	0.72–1.49		0.09	0.10	0.76	0.38	1.09	0.89–1.34	

**Figure 2:**
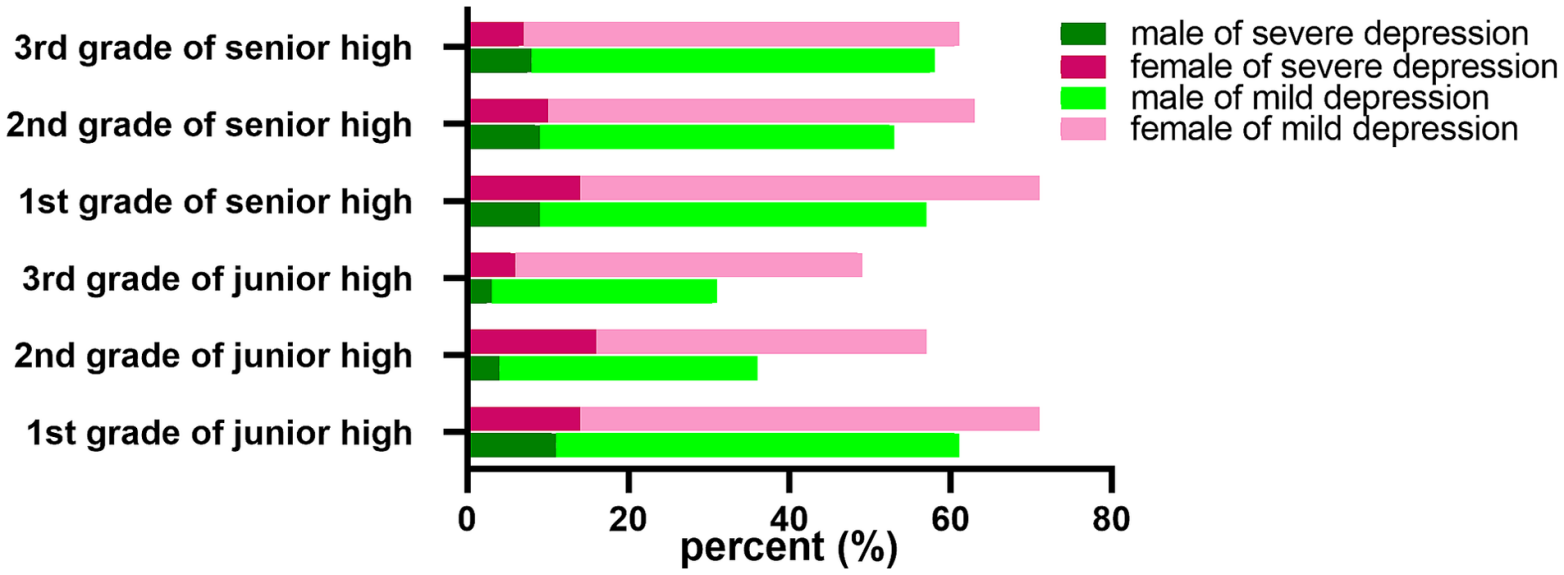
Differences in depressed mood according to grade and gender.

Moreover, a correlation analysis was performed between gender and grade (Figure 2). The number of boys in the high depression group and the low depression group was 0.54 times and 0.61 times that of girls, respectively. Pearson’s chi-square test showed that the detection rate of depression in girls (53.1%) was significantly higher than that in boys (42.7%) (*p* < 0.001). Stratified by grade, the prevalence of high depression in girls (16.5%) was significantly higher than that in boys (4.7%) in the 2^nd^ grade of junior middle school students group, with an OR value of 4.06 (95% CI, 2.241–7.386), *p* < 0.001. In the the 1^st^ grade of high school group, the detection rate of high depression was significantly higher in girls (14.5%) than in boys (9.6%), with an OR of 1.60 (95% CI, 1.059–2.406), *p* < 0.05.

### 3.4 Correlation between school category and depressed mood

**Figure 3:**
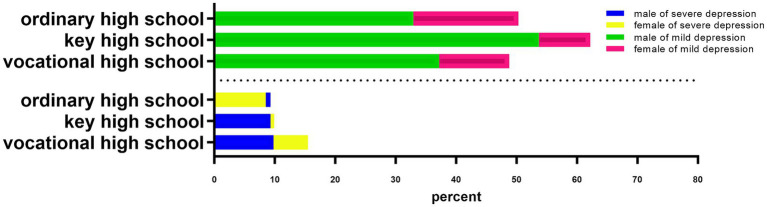
Differences in depressed mood according to school type.

Subsequently, a one-way logistic regression analysis was performed on the school category. As shown in Table 6, adolescents in vocational schools entered the high and low depression groups 1.73 and 1.21 times more than those in general secondary schools, respectively. Students in key secondary schools entered the high depression group and the low depression group 1.61 times and 2.07 times more than those in general secondary schools, respectively. As shown in Figure 3, the detection rate of the high depression group of vocational school adolescents was 13.5%, which was significantly higher than that of key secondary schools (9.6%) and general secondary schools (9.0%). The detection rate of 58.5% in the low depression group of students in key secondary schools was significantly higher than that in vocational secondary schools (44.7%) and general secondary schools (42.7%).

**Table 6:** Unordered multifactorial logistic regression of school type on potential class.

Variables			Severe depression (*N* = 316)							Mild depression (*N* = 1,465)					
		B	SE	Wald	P	OR	CI(95%)		B	SE	Wald	P	OR	CI(95%)	
School Type (General High School)	Key high school	0.48	0.22	4.53	0.03	1.61	1.04–2.50		0.73	0.13	30.99	0.00	2.07	1.60–2.68	
	Vocational High School	0.55	0.22	6.06	0.01	1.73	1.12–0.68		0.19	0.13	2.06	0.15	1.21	0.93–1.58	

## 4 Discussion

Adolescent depression is a psychological disorder with severe intermittent effects that include low mood, anxiety, and negative and low estimates of self-perception. It can affect adolescents’ behavior, mood, thinking, development, learning ability, and social interaction skills. Adolescent depression is becoming a social concern, stemming from its increasing incidence. The cumulative incidence of adolescent depression gradually increased from 5 to 20% during early adolescence (21). Depression is diagnosed in 13.0% of people aged 12–17 in the United States (22, 23). In this study, depression in adolescents aged 13–18 years was divided into three groups using latent profile analysis, with 10.1% in high depression, 48.4% in low depression, and 41.5% in no depression.

In the present study, a critical value of 14.5 (Se = 0.98, Sp = 1.00) for the PHQ-9 was used for the discrimination of depression. Cassiani-Miranda et al. suggested that the PHQ-9 was also found to be more reliable when used to screen for depression (24). A recent study suggested that the PHQ-9 with a critical score of 10.0 had the best sensitivity (Se = 0.91) and specificity (Sp = 0.76) among adolescents aged 10–19 years (25). An intervention study showed that cutpoints with PHQ-9 ≥ 17 provided 96% specificity and 16% sensitivity (26). However, the choice of different thresholds in these studies also influenced the evaluation results. Latent profile analysis (LPA) was used to identify depression subgroups among 1831 older adults living alone and classified as low (30.4%), moderate (55.3%), and high-level (14.4%) (27). Park et al. (28) explored the characteristics of self-esteem, life satisfaction, and depression, resulting in the identification of five subgroups, namely, extreme depression, major depression, mild depression, low life satisfaction, and positive adjustment. In most studies, depression’s standard symptoms were used as latent class indicators. Therefore, the PHQ-9 combined with the LPA can be used as a depression screening tool to identify depression early so that effective treatment can be provided in time.

In this study, the detection rate of the high depression group was high in 1st grade of junior middle school and 1st grade of high school. Tang et al. found depression detection rates of 40.1 and 33.0% in 2nd and 3rd grade of high school group, respectively (6). In another study, clinical depression detection rates among 12-14-year-olds were highest among 12-21-year-olds (15). Öngen et al. concluded that there were no significant differences in cognitive emotion regulation and depression across grades (29). It has been demonstrated that the prevalence of depressive symptoms decreases from junior middle school 3^rd^ grade to 1^st^ grade (9). A high prevalence of Internet gaming disorder has been observed in first-year secondary school students (30). Although these studies have shown a high prevalence of depression among adolescents, different studies have shown varying grades of students with higher prevalence of depression, which may be related to the timing of the survey. The survey period in this study is from October to November, which coincides with the first grade students just entering their new school, which is consistent with the time point of Chi’s research (9). The pressure faced by first-year students who have just entered a new environment may stem from interpersonal difficulties, as most classmates are unfamiliar and require good social skills to gain peer support and difficulties in determining their position in the new social hierarchy (30–32). These pressures are believed to be related to the onset of depression (33). Therefore, early intervention in the mental health management of new students is necessary for secondary schools.

This study showed that there were more girls than boys in both the high depression group and the low depression group. The detection rate of depression was significantly higher in 2^nd^ grade of junior middle school and the 1^st^ grade of high school girls than in boys. Studies have shown that the rate of depression detection among girls increases substantially during adolescence (1). Gender differences in depression detection rates appear after age 13 (1). Sun et al. also found gender differences in the detection of depression in adolescents between the ages of 12 and 15 (5). Depression detection rates rise in early adolescence in females and late adolescence in males (5). This phenomenon may be related to estrogen levels. The peak of menarche in Chinese girls is at the age of 12–13 years (34). Studies have shown that the relationship between estrogen levels and depression is strongly correlated with early development (35). Elevated estrogen levels make girls more sensitive to stress (21). Similarly, estrogen has been shown to increase the stress response in the prefrontal cortex in animal studies (36). Thus, the psychological changes of adolescent girls need to be focused on.

Boys scored higher in the following items: “Little interest or pleasure in doing things” and “Trouble concentrating “. The theory of gender intensification suggests that changes in the body are conducive to the development of gender roles (37). Boys focus on self-worth, while girls focus on relationships (38). A boy’s sense of self-worth comes from experiencing and exploring, and this can be seen through his interest and concentration (39, 40). Therefore, adolescent mental health management needs to consider more gender factors.

This study found that the prevalence of high depression in vocational schools was higher than that in key middle schools and general middle schools. Vocational students are more likely to suffer from mental illness (41–43). Firstly, this may be because vocational students face more chronic stress (14, 44). Ethnic minorities, single-parent families, poverty, and low educational levels of parents are the negative factors in the growth environment of adolescents (45–47). Students in vocational secondary schools face at least one negative factor; one accounts for 35.2%, two accounts for 13.49%, three accounts for 3.09%, and four accounts for 0.23% (14). Chronic stress stimulation has been shown in studies to decrease HPA axis activity, and HPA axis dysfunction is one of the pathogenesis factors of depression (48, 49). Vocational school students are often vulnerable groups with poor academic performance in many countries and regions (50). Students in vocational secondary schools have low satisfaction with school, are unable to adapt to the learning environment, and cannot face the frustration of selection tests (51). Therefore, students in vocational schools have a high probability of psychological problems and need to strengthen their mental health management.

This study used large sample data and latent profile analysis to determine a critical value of 14.5 for the PHQ-9 scale when applied to the population aged 13 to 18, which can provide recommendations for depression screening in middle school students. Our study also had some limitations, as unmeasured potential factors such as family environment and peer pressure might affect the research results. The sample was limited to six schools, and the representativeness and generalizability of the findings may be limited. The sample was a cross-sectional study, and a follow-up study may better illustrate the changes in depression during secondary school.

## 5 Conclusion

By analyzing the demographic, social, and environmental characteristics, this study found that adolescents aged 13 and 16 (the 1^st^ grade of junior middle school and the 1^st^ grade of high school students) were more likely to fall into depressive conditions, with girls requiring more attention than boys. Vocational school students face more pressure and need to strengthen their psychological guidance. Using this study as a basis, future research would be worthwhile to collect characteristics of symptom changes in the adolescent depression group from longitudinal studies.

## Data availability statement

The raw data supporting the conclusions of this article will be made available by the authors, without undue reservation.

## Ethics statement

The studies involving humans were approved by the Ethics Committee of the Hainan Provincial Anning Hospital. The studies were conducted in accordance with the local legislation and institutional requirements. Written informed consent for participation in this study was provided by the participants' legal guardians/next of kin.

## Author contributions

JZ contributed to funding the acquisition, investigation, and funding the acquisition. DL and LD contributed to the investigation. GD contributed to project administration, resources, writing-original draft, and writing review & editing. All authors contributed to the article and approved the submitted version.
